# Magnitude of Extended-Spectrum Beta-Lactamase-Producing Gram-Negative and Beta-Lactamase-Producing Gram-Positive Pathogens Isolated from Patients in Dar es Salaam, Tanzania: A Cross-Sectional Study

**DOI:** 10.7759/cureus.24451

**Published:** 2022-04-24

**Authors:** Doreen Mloka, Raphael Z Sangeda, Kennedy D Mwambete, Appolinary. R Kamuhabwa

**Affiliations:** 1 Department of Pharmaceutical Microbiology, Muhimbili University of Health and Allied Sciences, Dar es Salaam, TZA; 2 Department of Clinical Pharmacy and Pharmacology, Muhimbili University of Health and Allied Sciences, Dar es Salaam, TZA

**Keywords:** beta-lactamase enzymes, extended-spectrum beta-lactamase bacteria, esbl, tanzania, dar es salaam, antibiotic resistance, cephalosporins

## Abstract

Background

The worldwide emergence of antibiotic-resistant bacteria threatens to overshadow the dramatic advances in medical sciences since the discovery of antibiotics. Antibiotic resistance has rendered some antibiotics obsolete, creating a reliance on synthetic drugs. In some instances, bacteria can be resistant to all antibiotics. The problem of antibiotic resistance is eminent in resource-limited countries like Tanzania, where systematic surveillance and routine susceptibility tests are rarely conducted. Therefore, this study aimed to investigate the magnitude of beta-lactamase-producing Gram-positive pathogens and Enterobacteriaceae with extended-spectrum beta-lactamase (ESBL) in Dar es Salaam, Tanzania.

Methodology

This multi-site cross-sectional study involved three private hospitals in Dar es Salaam, Tanzania. The study was conducted between July and September 2008. Bacterial isolates were collected, identified, and subjected to antibiotic-sensitivity testing against cephalosporins, including ceftriaxone, cefuroxime and cefotaxime, and clavulanic acid, which are antibiotics readily available on the Tanzanian market at the time of the study. The microdilution method was employed to determine beta-lactamase and ESBL production per the Clinical Laboratory and Standards Institute (CLSI) protocol. Cephalosporins, including ceftriaxone, cefuroxime and cefotaxime, the beta-lactamase inhibitor, and clavulanic acid, were serially diluted with concentrations ranging from 0.011 mg/ml to 200 mg/ml. Each of these antibiotics was subjected to sensitivity tests by determining the minimum inhibitory concentrations (MIC) of the clinical isolates of bacteria using a 96-well microdilution plate. Five microliters of bacterial suspension were inoculated into each well-containing 120µl of sterile Mueller-Hinton broth before incubation overnight.

Results

A total of 111 bacterial isolates were tested. Of the 111 tested bacterial isolates, 85 (76.6%) and 26 (23.4%) were Gram-negative and Gram-positive bacteria, respectively. Fifty-six clinical isolates (50.4%) were *Escherichia coli, and* 13 *Salmonella *species (11.7%) were among the Gram-negative isolates. On the other hand, 15 (13.5%) and 11 (9.9%) Gram-positive bacteria were *Staphylococcus aureus* and *Streptococcus *species, respectively, of all isolates. The majority of these clinical isolates, 71 (64.0%), were obtained from mid-stream urine, while the remaining were from stool, vaginal secretions, blood, pus, catheter sip, and urethra. A high proportion of tested Gram-negative bacteria, 58 (68.2%), were identified as ESBL producers, and 16 (61.5%) of the Gram-positive bacteria were identified as beta-lactamase producers. Cefuroxime was the least effective, exhibiting the largest MIC (18.47 ± 22.6 mg/ml) compared to clavulanic acid alone (5.28 ± 8.0 mg/ml) and clavulanic acid-cefuroxime (5.0± 12.32 mg/ml). Of all isolates, 78.2% were sensitive to chloramphenicol. Only five isolates had MIC larger than 32.23 mg/ml as opposed to cefotaxime and ampicillin, which had more isolates in that similar MIC range.

Conclusion

There is a high proportion of beta-lactamase, particularly ESBL-producing pathogens, in Dar es Salaam, Tanzania. Therefore, regular detection of beta-lactamase and ESBL production may help detect resistance to beta-lactam antibiotics.

## Introduction

Antibiotic resistance is currently a worldwide health concern. It is worrisome when human pathogens become increasingly resistant to multiple antibiotics [1]. According to a new estimate, more than a million people died from bacteria-resistant infections worldwide in 2019, higher than malaria or AIDS numbers [1]. This situation indicates a silent pandemic that requires serious intervention [2]. Apparently, these death rates due to antibiotic-resistant infections are highest in sub-Saharan Africa [1]. Consequently, it has become more important for doctors and other health care providers to determine susceptibility and resistance to antibiotic agents in treating infections. Furthermore, it is even more important for researchers to develop new and more powerful classes of antibiotics to prevent and treat these infections.

In the last few years, members of the Enterobacteriaceae with extended-spectrum beta-lactamase (ESBL) have been identified. They also pose significant health threats worldwide [3-7]. Indeed, the emergence of ESBL-producing pathogenic Enterobacteria poses a serious antibiotic management problem since the ESBL genes are easily transferred from one organism to the other via plasmids. It is thus not surprising that these ESBL-containing organisms, which were hitherto found mainly in hospitals, are now becoming fairly common in community-acquired infections, especially those of the urinary tract [8-10]. Resistance to one of the extended-spectrum cephalosporins (ceftazidime, cefotaxime, or ceftriaxone), when mediated by an ESBL, may imply resistance to all, even when sensitivity test results might indicate otherwise [11]. Clinical microbiology laboratories are encouraged to adopt the Clinical Laboratory and Standards Institute (CLSI) guidelines to identify beta-lactamase and ESBL-producing organisms and institute appropriate therapy.

Studies conducted between 2001 and 2020 in Tanzania show a substantial proportion of ESBL-producing isolates in ICUs in some hospitals and some fatal cases of pediatric septicemia due to ESBL producers [3,12,13]. In Tanzania, the prevalence of ESBL producers could be aggravated by the fact that the majority of the livestock, and particularly poultry keepers, use antibiotics to treat their flock whenever they get ill before seeking other specialized veterinary care [5,14].

Equally important, the rates of Gram-positive resistant isolate resistance to antibiotics are also threatening the success of treating infectious diseases. Methicillin-resistant Staphylococcus aureus (MRSA) and other beta-lactamase-producing pathogens such as Streptococci species are among the culprits [15].

Generally, routine microbiological documentation of infections is practically impossible in our clinical settings due to limited financial and laboratory testing capacity. Hence knowledge of the actual microbial cause of a particular infection and its susceptibility to antibiotics, which is essential for the rational use of antibiotics, is amiss. This is worsened by widespread counterfeit medicines and inappropriate use of antibiotics [16,17], which might have contributed to the emergence of drug resistance, particularly by the beta-lactamase and ESBL pathogenic bacteria [3,13]. Therefore, this study assessed beta-lactamase Gram-positive and ESBL Gram-negative bacterial isolates and compared their susceptibility patterns to commonly used antibiotics.

## Materials and methods

Study design

This was a cross-sectional study that involved the collection of clinical isolates of bacteria from three major private hospitals, namely Regency Medical Centre, Shree Hindu Mandal, and Massana Hospitals, located within the Dar es Salaam region in Tanzania from July to September 2008.

Collection of bacterial isolates

All samples identified bacteria from old plates that were available after use at the laboratories of each respective hospital during the study period. They were screened for evidence of potential beta-lactamase or ESBL production according to the CLSI protocols [10,18]. Samples included urine, feces, blood, pus, vaginal secretions, and other body fluids. The collected isolates were transported to our research laboratory of Pharmaceutical Microbiology at Muhimbili University of Health and Allied Sciences (MUHAS) using a routine transport medium (Peptone Water, Oxoid, UK). Three strains of E. coli (ATCC 25922), Pseudomonas aeruginosa (ATCC 27853), and Staphylococcus aureus (ATCC 25923), obtained from the Laboratory of Microbiology and Immunology, MUHAS were employed as reference standard bacteria and negative control of beta-lactamase and ESBL production [11].

Identification and preparation of clinical isolates of bacteria

All sampled clinical isolates were subjected to microbiological identification tests based on colony growth characteristic morphologies and biochemical tests [19]. Each microorganism was sensitized by sub-culturing in freshly prepared Mueller-Hinton broth (Roth, Germany), followed by overnight incubation at 37°C. The turbidity of each bacterial suspension was matched to that of McFarland 0.5 standard turbidity (equivalent to 5×106 colony forming units/ml) before performing antibiotic activity assays [10].

Screening for beta-lactamase and ESBL producers

Serially diluted solutions of cephalosporin antibiotics, including ceftriaxone, cefuroxime, cefotaxime, and a beta-lactamase inhibitor (clavulanate), with concentrations ranging from 0.011 mg/ml to 200 mg/ml which were in the range of therapeutic doses of injectable cephalosporins, were prepared. Each of these antibiotics was subjected to sensitivity tests using the minimum inhibitory concentration (MIC) determination method on the clinical isolates of the identified bacteria using a 96-well microdilution plate. Five microliters of bacterial suspension were inoculated into each well-containing 120 µl of sterile Mueller-Hinton broth (Roth-German) [10], incubated overnight, and inspected for visible turbidity.

Interpretation of beta-lactamase and ESBL production

Confirmation of beta-lactamase or ESBL production involved the determination of MICs for a combination of ceftriaxone-clavulanate, cefuroxime-clavulanate, and cefotaxime-clavulanate, compared with MIC obtained from each cephalosporin alone. A three-fold or greater difference of MIC corresponding to a decrease of ≥3 serial dilutions in a MIC for either ceftriaxone or cefuroxime tested in combination with clavulanic acid versus when tested alone confirmed the production of beta-lactamase or ESBL [20,21].

Comparative susceptibility pattern testing of ESBL and non-ESBL producers

All ESBL and non-ESBL isolates were subjected to susceptibility pattern testing against cefotaxime, ampicillin (Glaxo, Italy), and chloramphenicol (Alkem Lab., India) (unaffected by ESBLs) using the same method through which their MICs were compared. The control microorganisms were also tested in this assay. The choice of tested antibiotics is based largely on their easy availability in injectable and wide application in the Tanzania market at the time of the study.

Statistical data analysis

All the procedures above were performed twice for statistical purposes and consistent results. Therefore, the MICs were expressed as mean, and data were entered into the statistical package. Statistical analysis was done using the Statistical Package for the Social Sciences (SPSS 15.0.2006) software (SPSS Inc., Chicago, IL). Differences in MICs among different isolates and reference strains of bacteria at various concentrations were compared and considered significant when the p-value < 0.05.

Ethical clearance

The ethical clearance to conduct the study was provided by the Muhimbili University of Health and Allied Sciences (MUHAS) Institutional Review Board (IRB). Administrative permissions were obtained from the relevant hospital authorities. All clinical isolates of bacteria were coded such that neither names nor personal information were revealed to endanger patients' confidentiality.

## Results

Prevalence of ESBL Gram-negative and beta-lactamase Gram-positive-producing bacteria

Clinical isolates of bacteria were collected from three major private hospitals in Dar es salaam, Tanzania. A total of 111 bacterial isolates from clinical specimens collected were tested and compared with three reference strains of bacteria. Of 111 tested bacterial isolates, 85 (76.6%) and 26 (23.4%) were Gram-negative and Gram-positive bacteria, respectively. Most, 71 (64.0%), of the clinical isolates were from mid-stream urine, of which E. coli constituted 49.6% (Table 1). Of 111 clinical isolates, the majority, 56 (50.4%), were E. coli, and the minority, 2 (1.8%), were Pseudomonas species (Table 1).

**Table 1:** Distribution of specimens' sources of tested Gram-negative and Gram-positive bacterial isolates. ECO: *Escherichia coli*; STA: *Staphylococcus aureus*; SAL: *Salmonella species*; KLE: *Klebsiella species*; PRO: *Proteus species*; PSE: *Pseudomonas species*; STR: *Streptococcus species.*

	Bacterial isolates n(%)						
	Gram-negative (N = 85)				Gram-positive (N = 26)		
Source	ECO	KLE	PSE	SAL	STA	STR	Source total
Urine	55 (49.6)	7 (6.3)	2 (1.8)	0 (0)	3 (2.7)	3 (2.7)	71 (64.0)
Stool	0 (0)	0 (0)	0 (0)	13 (11.7)	0 (0)	2 (1.8)	15 (13.5)
Vaginal secretions	0 (0)	0 (0)	0 (.00)	0 (0)	2 (1.8)	2 (1.8)	7 (6.3)
Pus	0 (0)	0 (0)	0 (.00)	0 (0)	9 (8.1)	1 (0.90)	12 (10.81)
Blood	0 (0)	0 (0)	0 (.00)	0 (0)	0 (0)	3 (2.7)	3 (2.7)
Catheter sip	1 (0.90)	0 (0)	0 (.00)	0 (0)	0 (0)	0 (0)	2 (1.8)
Urethra	0 (0)	0 (0)	0 (.00)	0 (0)	1 (0.90)	0 (0)	1 (0.9)
Specie total	56 (50.4)	7 (6.3)	2 (1.8)	13 (11.7)	15 (13.5)	11 (9.9)	111 (100)

**Figure 1:**
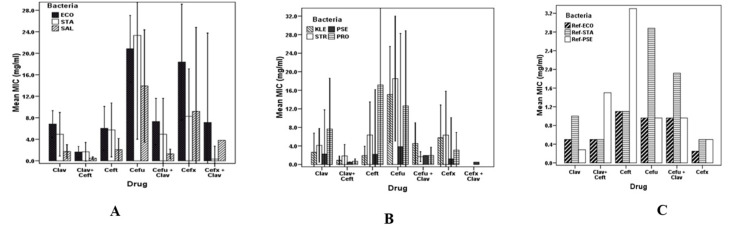
Susceptibility patterns of clinically tested isolates of bacteria to cephalosporins. Panel A: *Escherichia coli*, *Staphylococcus aureus, *and *Salmonella *species. Panel B: *Streptococcus*, *Pseudomonas, *and *Proteus *species. Panel C: Strains of reference bacteria strains. ECO: *Escherichia coli*; SAL: *Salmonella *species; KLE: *Klebsiella *species; PRO: *Proteus *species; PSE: *Pseudomonas *species; STA: *Staphylococcus aureus*; STR: *Streptococcus *species*.*

Of all tested cephalosporins, cefuroxime was the least effective, with the mean of MICs, 18.47±22.6 mg/ml versus 5.28±8.0 mg/ml and 5.0± 12.32 mg/ml, exhibited by clavulanic acid alone and clavulanic acid-cefuroxime combination, respectively. Isolates of E. coli and Proteus species displayed the highest mean of MIC. There were no statistically significant differences (df 9; F = 1.38; p-value = 0.194) among the clinical isolates of bacteria with respect to MIC by neither the rest of the individual antibiotics nor their combinations with clavulanic acid depicted in Figure 1 (Panels A and B). Statistically significant differences in MIC (p-value < 0.05) were observed between strains of reference bacteria and clinical isolates (Figure 1).

On one hand, 58 (68.2%) of the Gram-negative isolates were identified as ESBL producers. On the other hand, 16 (61.5%) of the Gram-positive bacteria were classified as beta-lactamase producers. The reference strains of bacteria used were negative for ESBL or beta-lactamase production, wherein E. coli (ATCC 25922) served as a negative control microorganism (CLSI, 2006). Out of 58 isolates, nine clinical isolates of Proteus species had the highest proportion of ESBL (85.7%) of that species isolates. The proportion of ESBL in other Gram-negative bacteria were *Klebsiella* species (71.4%), *Salmonella* species (69.2%), *E. coli* (66.1%), and *Pseudomonas* species (50.0%) (Tables 2).

**Table 2:** Total number of Gram-negative extended-spectrum beta-lactamase or Gram-positive beta-lactamase producers with respect to their species and sources. ECO: *Escherichia coli*; SAL: *Salmonella* species; KLE: *Klebsiella* species; PRO: *Proteus* species; PSE: *Pseudomonas* species; STA: *Staphylococcus aure*us; STR: *Streptococcus* species.

	Percentage of each bacterial specie								
Source	Gram-negative extended-spectrum beta-lactamase producers n (%)						Gram-positive beta-lactamase producers n (%)		
	ECO (n=56)	KLE (n=7)	PSE (n=2)	PRO (n=7)	SAL (n=13)	Total Gram negative (n=85)	STA (n=15)	STR (n=11)	Total Gram positive (n=26)
Urine	35 (62.5)	5 (71.4)	1 (50.0)	2 (28.6)	0 (0.0)	43 (55.6)	2 (13.2)	1 (9.1)	3 (11.5)
Stool	0 (0.0)	0 (0.0)	0 (0.0)	0 (0.0)	9 (69.2)	9 (10.6)	0 (0.0)	1 (9.1)	1 (3.8)
Vaginal secretions	0 (0.0)	0 (0.0)	0 (0.0)	2 (28.6)	0 (0.0)	2 (2.3)	1 (6.7)	1 (9.1)	2 (7.7)
Pus	0 (0.0)	0 (0.0)	0 (0.0)	2 (28.6)	0 (0.0)	2 (2.3)	5 (33.3)	1 (9.1)	6 (23.1)
Blood	0 (0.0)	0 (0.0)	0 (0.0)	0 (0.0)	0 (0.0)	0 (0.0)	0 (0.0)	4 (36.4)	4 (15.4)
Catheter sip	2 (3.6)	0 (0.0)	0 (0.0)	0 (0.0)	0 (0.0)	2 (2.3)	0 (0.0)	0 (0.0)	0 (0.0)
Specie total	37 (66.1)	5 (71.4)	1 (50.0)	6 (85.7)	9 (69.2)	58 (68.2)	8 (53.3)	8 (72.7)	16 (61.5)

Gram-positive bacteria exhibited high MIC values (Figure 1 and Table 2). An apparent increase of MIC among the assayed cephalosporins alone and with a beta-lactamase inhibitor (clavulanic acid) was demonstrated by the ESBL producers exhibiting an about 40-fold increase of MIC (Figure 1). High MIC variations are seen in clinical isolates from varying sources than in the MICs of the reference bacterial strains.

Susceptibility of beta-lactamase and non-beta-lactamase bacteria to non-beta-lactam antibiotics

**Figure 2:**
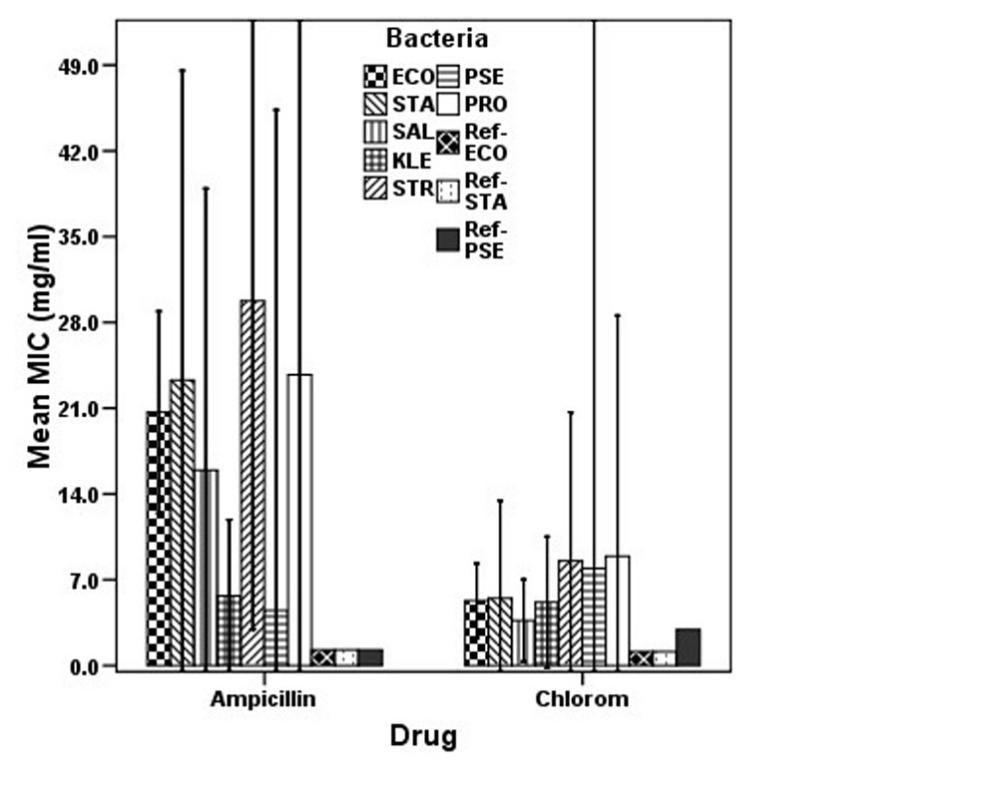
Susceptibility profiles of bacterial isolates to two most commonly used antibiotics. ECO: Escherichia coli; STA: Staphylococcus aureus; SAL: Salmonella species; KLE: Klebsiella species; PRO: Proteus species; PSE: Pseudomonas species; STR: Streptococcus species; R: Reference bacteria strains.

When comparing the sensitivity of beta-lactamase and non-beta-lactamase-producing isolates to chloramphenicol (non-beta-lactam antibiotics), there were significant differences (df = 2, F = 7.614; p<0.001) regarding MIC among the two groups (Table 3). Chloramphenicol was relatively effective compared to cefotaxime and ampicillin, while the strains of reference bacteria were equally sensitive (p-value < 0.05) to all antibiotics tested (Figure 2). The majority (78.2%) of the assayed isolates were susceptible to chloramphenicol. Only five isolates had MIC greater than 32.23 mg/ml as opposed to cefotaxime and ampicillin, which had more isolates in that similar MIC range (Table 3). Of all isolates, S. aureus and Proteus species were the most resistant to chloramphenicol and ampicillin, yielding MIC of 150 mg/ml and 64 mg/ml, respectively.

**Table 3:** Sensitivity of both Gram-negative and Gram-positive bacterial isolates to antibiotics measured using minimum inhibitory concentration test. ECO: Escherichia coli; STA: Staphylococcus aureus; SAL: Salmonella species; KLE: Klebsiella species; PRO: Proteus species; PSE: Pseudomonas species; STR: Streptococcus species; R: Reference bacteria strains.

Antibiotic	MIC range	Number of bacteria isolates										Total
		ECO	STA	SAL	KLE	STR	PSE	PRO	R-ECO	R-PSE	R-STA	
Cefotaxime	≤0.14	4	1	1	0	1	0	0	0	0	0	7
	0.15-4.15	31	11	9	6	8	2	5	1	1	1	75
	4.16-8.16	1	1	2	0	0	0	1	0	0	0	5
	8.17-12.17	6	0	0	0	1	0	1	0	0	0	8
	20.20-24.20	6	0	0	1	0	0	0	0	0	0	7
	≥32.23	7	2	1	0	1	0	0	0	0	0	11
	Total	55	15	13	7	11	2	7	1	1	1	113
Chloramphenicol	≤0.14	8	1	2	0	ND	0	2	0	0	0	14
	0.15-4.15	34	11	9	5	ND	1	4	1	1	1	75
	4.16-8.16	4	2	0	0	ND	0	0	0	0	0	6
	12.18-16.18	6	0	2	2	ND	1	0	0	0	0	11
	28.22-32.22	1	0	0	0	ND	0	0	0	0	0	2
	≥32.23	2	1	0	0	ND	0	1	0	0	0	5
	Total	55	15	13	7	ND	2	7	1	1	1	113
Ampicillin	≤0.14	6	0	3	0	0	0	0	0	0	0	9
	0.15-4.15	21	10	8	5	4	1	4	1	1	1	56
	4.16-8.16	4	2	0	0	0	1	0	0	0	0	7
	12.18-16.18	9	0	0	2	4	0	2	0	0	0	17
	28.22-32.22	5	0	0	0	0	0	0	0	0	0	5
	≥32.23	10	3	2	0	3	0	1	0	0	0	19
	Total	55	15	13	7	11	2	7	1	1	1	113

Results also show significant differences in susceptibility to tested cephalosporins. Cefuroxime was the least effective in killing the bacteria, with mean MIC of 18 mg/ml and 24 mg/ml for S. aureus and Staphylococcus isolates, respectively (df = 9; F = 2.615; p-value = 0.006), as depicted in (Figure 2B-2C). Similarly, based on the source of specimens, significant differences in susceptibility (MIC) were observed among the isolates (df = 7; F = 4.533; p-value = 0.0001). These observations clearly show that chloramphenicol is relatively more susceptible, exhibiting MICs of 6 mg/ml and 8 mg/ml against Streptococcus species and S. aureus, respectively (Figures 1 and 2).

## Discussion

Beta-lactam antibiotics are among the most frequently prescribed antibiotics worldwide [17]. However, in the last few years, the emergence of resistance to these agents has become a major health problem [1,22], particularly in resource-limited countries like Tanzania [1,3,13]. Several factors have been ascribed to this situation, such as producing a variety of ß-lactamases that deactivate beta-lactam antibiotics, alterations in the penicillin-binding proteins, outer membrane permeability, and combinations of multiple mechanisms of resistance. This increase has paralleled the introduction, administration, and overuse of ß-lactam antibiotics [17]. However, the prevalence of ESBL-producing bacteria in most hospitals remains unknown despite numerous reports of nosocomial infection outbreaks due to these microorganisms. Important ESBL-producing bacteria include K. pneumoniae, E. coli, Proteus mirabilis, Enterobacter species, Citrobacter freundii, and P. aeruginosa, to cite a few [23].

Another study in Tanzania among poultry meat vendors in Dar es Salaam revealed that 35.7% of the surveyed population harbored ESBL-producing E. coli and Klebsiella species [24]. These ESBL organisms could easily spread from the vendors to the meat and, finally, to consumers in the food chain. ESBL organisms found in environmental samples along the Msimbazi River Basin Ecosystem in Tanzania were resistant to more than three classes of the antibiotics tested [5]. In another study in an urban study site in Keko Machungwa, part of the most significant unplanned and under-serviced settlement in Temeke district, Dar es Salaam, Tanzania, ESBL-producing Enterobacteriaceae were confirmed in 24.3% of the 70 latrine samples [7].

The fact that most (64%) of the tested bacterial isolates were derived from midstream urine and that majority (49.6%) comprised of E. coli is not uncommon since these are frequently implicated in UTIs [25]. Moreover, E. coli is isolated frequently because midstream urine is routinely done than other tests. Results also have revealed bacterial resistance, evidenced by high MICs, to both second and third lines cephalosporins, which are the antibiotics of choice for bacterial infections in Tanzania. Chloramphenicol has exhibited adequate effectiveness against the clinical isolates; this could be predictive of its revival since it was considered obsolete for several bacterial infections [26]. Immediate action needs to be taken to alleviate this situation.

The discrepancy in susceptibility observed among the bacterial isolates could be explained because they are distinct species with different mechanisms of counteracting drugs action [21]. Nevertheless, the fact remains that most of the Gram-negative isolates (68.2%) were identified as ESBL producers. This has economic implications; thus, efforts should be directed toward curbing the situation before spreading all over the country. Salmonella was the second most frequently isolated Gram-negative bacteria (11.7%). It is well known that Dar es Salaam has sanitary problems, accompanied by a lack of portable and safe drinking water and facilities for waste disposal. Thus typhoid fever and other water-borne bacterial infections are rampant. Inappropriate antibiotic usage [16,17] and poor sanitary conditions could have greatly contributed to the spread of antibiotic-resistant Salmonella isolates [7].

Our study revealed that 68.2% of the Gram-negative isolates were ESBL producers, while 61.5% of Gram-negative isolates were beta-lactamase producers. Of all the isolates sampled, the majority (50.4%) were E. coli. It is well recognized that E. coli's high agility in exchanging genetic information with various species is not uncommon. The increased presence of E. coli in nature can partly explain this finding [5]. The percentage of isolates expressing ESBL production is variable, as revealed by several previous studies: in the United States is 9.2%, Argentina (48%), France (11.4%), and India (68%), which is relatively similar to our findings. In these studies, the most frequently encountered ESBL producers were Gram-negative bacilli, Klebsiella species, and E. coli [3,4,13].

The relative frequency of ESBL producing Gram-negative bacilli in our study coincides with what was reported in Pakistan [27], where *E. coli *were also the predominant ESBL producing isolates (48%). Identification of *E. coli, P. aeruginosa, S. aureus* and *Klebsiella *species as potential ESBL producers in this study is of major concern for our country with the prevalence rate of approximately 7% of HIV-infected individuals [28]. Simply because some of these bacteria are multidrug-resistant and causative agents of opportunistic infections in immunocompromised individuals and critically ill patients in intensive care units [29]. Suppose this situation is not contained and appropriate measures are adopted. In that case, the country may be obliged to import more expensive antibiotics like carbapenems or cephamycins that appear to be relatively unaffected by ESBL producers, especially when they are used in combination with an aminoglycoside, fluoroquinolone, or ß-lactamase inhibitor [17,30]. Nevertheless, bacterial strains resistant to most classes of antibiotics will continue to emerge unless inappropriate use of these antibiotics is curtailed [31].

The study has some limitations, such as the small sample size used. In addition, the study relied on samples collected for routine hospital use rather than a systematic sampling of isolates in the city of study. Likewise, some bacteria were infrequently obtained from the clinical specimen sources, thus making the comparison uneven. The comparison could have been more appropriate had we had an equal number of bacterial isolates. Nevertheless, the study provides early evidence of the rising epidemic of ESBL and beta-lactamase-producing pathogens in Tanzania.

## Conclusions

We detected a higher proportion of beta-lactamase and particularly ESBL-producing pathogens with resistance to beta-lactam antibiotics among Gram-negative and Gram-positive bacteria from clinical isolates in Dar es Salaam. Therefore, tests for detecting beta-lactamase and ESBL production can be a quick way to spot these antibiotic-resistant pathogens.

## References

[1] Antimicrobial Resistance Collaborators (2022). Global burden of bacterial antimicrobial resistance in 2019: a systematic analysis. Lancet.

[2] Mahoney AR, Safaee MM, Wuest WM, Furst AL (2021). The silent pandemic: emergent antibiotic resistances following the global response to SARS-CoV-2. iScience.

[3] Ndugulile F, Jureen R, Harthug S, Urassa W, Langeland N (2005). Extended spectrum beta-lactamases among Gram-negative bacteria of nosocomial origin from an intensive care unit of a tertiary health facility in Tanzania. BMC Infect Dis.

[4] Abayneh M, Worku T (2020). Prevalence of multidrug-resistant and extended-spectrum beta-lactamase (ESBL)-producing gram-negative bacilli: a meta-analysis report in Ethiopia. Drug Target Insights.

[5] Kimera ZI, Mgaya FX, Mshana SE, Karimuribo ED, Matee MI (2021). Occurrence of extended spectrum beta lactamase (ESBL) producers, quinolone and carbapenem resistant Enterobacteriaceae isolated from environmental samples along Msimbazi river basin ecosystem in Tanzania. Int J Environ Res Public Health.

[6] Sonda T, Kumburu H, van Zwetselaar M, Alifrangis M, Lund O, Kibiki G, Aarestrup FM (2016). Meta-analysis of proportion estimates of Extended-Spectrum-Beta-Lactamase-producing Enterobacteriaceae in East Africa hospitals. Antimicrob Resist Infect Control.

[7] Erb S, D'Mello-Guyett L, Malebo HM (2018). High prevalence of ESBL-Producing E. coli in private and shared latrines in an informal urban settlement in Dar es Salaam, Tanzania. Antimicrob Resist Infect Control.

[8] Cotton MF, Wasserman E, Pieper CH (2000). Invasive disease due to extended spectrum beta-lactamase-producing Klebsiella pneumoniae in a neonatal unit: the possible role of cockroaches. J Hosp Infect.

[9] Burgess DS, Hall RG 2nd, Lewis JS 2nd, Jorgensen JH, Patterson JE (2003). Clinical and microbiologic analysis of a hospital's extended-spectrum β-lactamase-producing isolates over a 2-year period. Pharmacotherapy.

[10] Jonathan N (2005). Screening for extended-spectrum beta-lactamase-producing pathogenic enterobacteria in district general hospitals. J Clin Microbiol.

[11] Centers for Disease Control and Prevention (CDC) (2000). Laboratory capacity to detect antimicrobial resistance, 1998. MMWR Morb Mortal Wkly Rep.

[12] Navon-Venezia S, Hammer-Munz O, Schwartz D, Turner D, Kuzmenko B, Carmeli Y (2003). Occurrence and phenotypic characteristics of extended-spectrum β-lactamases among members of the family Enterobacteriaceae at the Tel-Aviv Medical Center (Israel) and evaluation of diagnostic tests. J Clin Microbiol.

[13] Blomberg B, Jureen R, Manji KP (2005). High rate of fatal cases of pediatric septicemia caused by gram-negative bacteria with extended-spectrum beta-lactamases in Dar es Salaam, Tanzania. J Clin Microbiol.

[14] Sangeda RZ, Baha A, Erick A (2021). Consumption trends of antibiotic for veterinary use in Tanzania: a longitudinal retrospective survey from 2010-2017. Front Trop Dis.

[15] Upreti N, Rayamajhee B, Sherchan SP, Choudhari MK, Banjara MR (2018). Prevalence of methicillin resistant Staphylococcus aureus, multidrug resistant and extended spectrum β-lactamase producing gram negative bacilli causing wound infections at a tertiary care hospital of Nepal. Antimicrob Resist Infect Control.

[16] Mwambete KD (2009). Irrational antibiotic usage in boarding secondary school settings in Dar es Salaam. East Afr J Public Health.

[17] Sangeda RZ, Saburi HA, Masatu FC (2021). National antibiotics utilization trends for human use in Tanzania from 2010 to 2016 inferred from Tanzania Medicines and Medical Devices Authority importation data. Antibiotics (Basel).

[18] (2022). Institute Clinical Laboratory Standards: Performance Standards for Antimicrobial Susceptibility Testing. https://clsi.org/standards/products/microbiology/documents/m100/.

[19] Balows A (2003). Manual of clinical microbiology 8th edition: P. R. Murray, E. J. Baron, J. H. Jorgenson, M. A. Pfaller, and R. H. Yolken, eds., ASM Press, 2003, 2113 pages, 2 vol, 2003 + subject &amp; author indices, ISBN: 1-555810255-4, US$ 189.95. Diagn Microbiol Infect Dis.

[20] Wormser GP, Kallen AJ (2002). Antimicrobial Pharmacodynamics in Theory and in Clinical Practice Edited by Charles H. Nightingale, Takeo Murakawa, and Paul G. Ambrose New York: Marcel Dekker, 2002. 416 pp., illustrated. $175.00 (hardcover). Clin Infect Dis.

[21] Livermore DM, Brown DF (2001). Detection of β-lactamase-mediated resistance. J Antimicrob Chemother.

[22] Rawat D, Nair D (2010). Extended-spectrum β-lactamases in Gram negative bacteria. J Glob Infect Dis.

[23] Sahni RD, Mathai D, Sudarsanam TD, Balaji V, Brahamadathan KN, Jesudasan MV, Lalitha MK (2018). Extended-spectrum beta-lactamase producers: detection for the diagnostic laboratory. J Glob Infect Dis.

[24] Mwanginde LW, Majigo M, Kajeguka DC, Joachim A (2021). High carriage rate of extended-spectrum β-lactamase-producing Escherichia coli and Klebsiella species among poultry meat vendors in Dar es Salaam: the urgent need for intervention to prevent the spread of multidrug-resistant pathogens. Int J Microbiol.

[25] Nester E, Anderson D, Salm S, Beins M (2011). Nester's Microbiology: A Human Perspective. AbeBooks - 0073375314. McGraw-Hill Science/Engineering/Math; 2011..

[26] Drago L (2019). Chloramphenicol resurrected: a journey from antibiotic resistance in eye infections to biofilm and ocular microbiota. Microorganisms.

[27] Shah AA, Hasan F, Ahmed S, Hameed A (2002). Extended-spectrum beta-lactamases in Enterobacteriaceae: related to age and gender. New Microbiol.

[28] (2019). United Republic of Tanzania: HIV/AIDS/STI SURVEILLANCE REPORT 24 (Tanzania Main Land 2013-2014). https://www.nacp.go.tz/download/hiv-aids-sti-surveillance-report-24-tanzania-main-land-2013-2014/..

[29] Aristizábal-Hoyos AM, Rodríguez EA, Arias L, Jiménez JN (2019). High clonal diversity of multidrug-resistant and extended spectrum beta-lactamase-producing Escherichia coli in a wastewater treatment plant. J Environ Manage.

[30] Artero A, Esparcia A, Alberola J, Madrazo M, Nogueira JM, Eiros JM (2017). Prospective cohort study of risk factors for extended-spectrum ß-lactamase-producing Escherichia coli urinary tract infections in elderly patients admitted to hospital. Int J Clin Pract.

[31] Abushaheen MA, Muzaheed Muzaheed, Fatani AJ (2020). Antimicrobial resistance, mechanisms and its clinical significance. Dis Mon.

